# Immunoglobin and T cell receptor repertoire changes induced by a prototype vaccine against Chagas disease in naïve rhesus macaques

**DOI:** 10.21203/rs.3.rs-3453582/v1

**Published:** 2023-11-02

**Authors:** Eric Dumonteil, Weihong Tu, Hans Desale, Kelly Goff, Preston Marx, Jaime Ortega-Lopez, Claudia Herrera

**Affiliations:** Tulane University; Tulane University; Tulane University; Tulane University; Tulane university; CINVESTAV; Tulane University

**Keywords:** Trypanosoma cruzi, TCR, IgG, CDR3 domain, immune response, RNA sequencing

## Abstract

A vaccine against Trypanosoma cruzi, the agent of Chagas disease, would be an excellent additional tool for disease control. A recombinant vaccine based on Tc24 and TSA1 parasite antigens was found to be safe and immunogenic in naïve macaques. Here we performed a transcriptomic analysis of PBMC responses to vaccination, to shed light on the immunogenicity of this vaccine and guide the optimization of doses and formulation. RNA-sequencing analysis indicated a clear transcriptomic response of PBMCs from macaques after three vaccine doses, with the up-regulation of several immune cell activation pathways and a broad non-polarized immune profile. Analysis of the IgG repertoire showed that it had a rapid turnover with novel IgGs produced following each vaccine dose, while the TCR repertoire presented several persisting clones that were expanded after each vaccine dose. These data suggest that three vaccine doses may be needed for optimum immunogenecity and support the further evaluation of the protective efficacy of this vaccine.

## INTRODUCTION

Chagas disease is a zoonotic parasitic disease of the Americas, caused by the protozoan parasite *Trypanosoma cruzi*, and transmitted primarily by hematophagous triatomine bugs. Disease burden reaches at least 6 millions cases in Latin America alone ^1^ and a global burden of 806,170 Disease-adjusted life years (DALYs) and an associated $627.46 million in health-care costs ^2^.

The disease starts with a short acute phase lasting a few weeks with flu-like signs and symptoms, followed by a chronic phase initially asymptomatic, but 30–40% of infections will progress to symptomatic cardiac or digestive disease, sometimes decades after infection ^3^. Only two drugs are available for treating patients, but their efficacy decreases dramatically as disease progresses, and their side effects lead to frequent treatment interruptions and non-compliance ^4–6^. Structural barriers for accessing diagnostic testing and treatment also considerably restrict access to appropriate care for patients ^7–9^.

Because of the limitations of current drug treatments, a vaccine would be an excellent additional tool for disease control. Many studies have shown the feasibility of multiple vaccine antigens and platforms in mice ^10,11^, and a few vaccine candidates have been tested in other animal models including dogs and non-human primates. Among these, a therapeutic DNA vaccine based on Tc24 and TSA1 parasite antigens has been shown to be effective to prevent cardiac alterations caused by *T. cruzi* infection in experimentally-infected macaques ^12^. A recombinant protein version of this vaccine is undergoing further development ^13–15^ and was found to be safe and immunogenic in naïve macaques ^16^. Indeed, high titers of antibodies and antigen-specific cytokine production by T cells were detected following subcutaneous immunization with three doses of recombinant Tc24-C4 and TSA1-C4 antigens formulated with a TLR4 adjuvant, warranting a more detailed evaluation of the immune response induced by vaccination.

Here we performed a transcriptomic analysis of PBMC responses to vaccination in naïve rhesus macaques, to shed light on the immunogenicity of this Chagas disease vaccine and guide the optimization of vaccine doses and formulation. In particular, we focus on identifying IgG and T cell receptor repertoires, which can inform in detail on the breadth of the immune response induced by vaccination ^17–20^.

## RESULTS

### PBMC transcriptomic profile following vaccination

**Figure 1:**
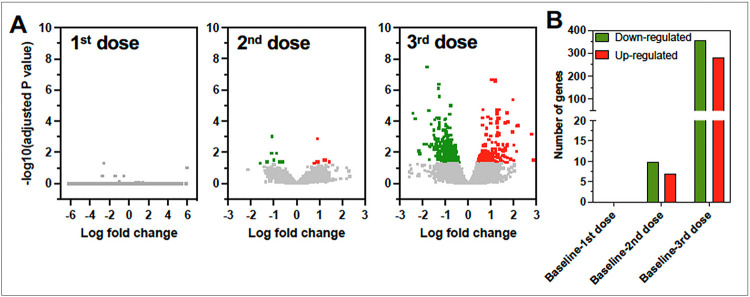
PBMC transcriptomic profile after vaccination **(A)** Volcano plots of gene expression levels after each vaccine dose compared to baseline expression levels. Gray dots indicate genes with no significant difference in expression levels, while colored dots indicate genes that are significantly up- (Red) and down- (Green) regulated compared to baseline expression level. **(B)** Counts of up- (Red) and down- (Green) regulated genes after each vaccine dose (Adjusted P value<0.05).

We first assessed the transcriptomic profile of PBMCs from vaccinated macaques one month after each of three vaccine doses, to further assess the immunogenicity of this candidate vaccine against *T. cruzi*. RNA-sequencing yielded 12–35 million quality reads/sample and over 80% were successfully mapped to the macaque genome. Differential expression analysis indicated that the gene expression profile of PBMCs was not significantly altered after the first vaccine dose, and only 17 genes were differentially expressed compared to baseline levels after the second vaccine dose. On the other hand, after the third vaccine dose, a total of 639 genes were differentially expressed, with 283 that were up-regulated and 356 down-regulated (Figure 1).

**Figure 2:**
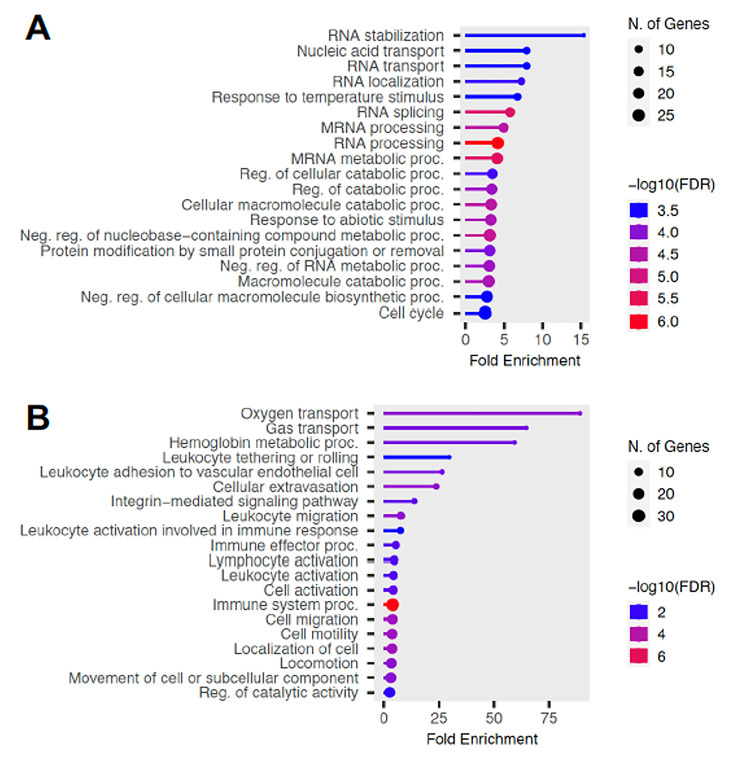
Biological pathway analysis of differentially expressed genes. Top 20 functional pathways associated with down- **(A)**and up- **(B)** regulated genes after vaccination (3 doses) based on GO Biological Process database. Circle size is proportional to the number of genes from the respective pathways that are differentially expressed and FDR-adjusted P values are color-coded as indicated.

Pathway analysis was performed based on differentially expressed genes after the third vaccine dose. Biological pathways associated down-regulated genes following vaccination included multiple RNA processing pathways as well as several metabolic/catabolic pathways (Figure 2A). On the other hand, up-regulated genes were associated with leukocytes and lymphocytes activation pathways and other immune system activation, as well as cell migration, adhesion or activation (Figure 2B). These data suggested a strong activation of PBMCs and of the immune response after the three vaccine doses.

**Figure 3:**
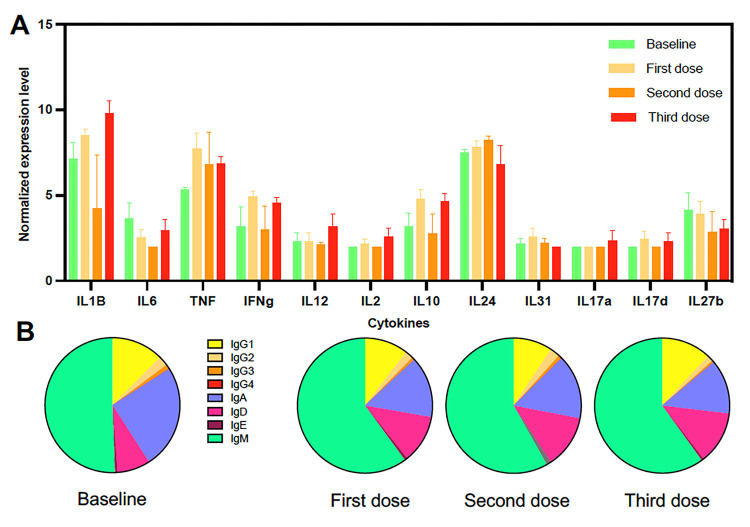
cytokine and antibody expression profile induced by vaccination. **(A)** Cytokine expression profile is shown after each vaccine dose. Data are presented as mean ±SEM for the indicated cytokines, which cover some of the Th1, Th2, and Th17 cytokines. **(B)** Ig subclass proportion after each vaccine dose. The subclasses are color coded as indicated.

For a better understanding of the orientation of the immune response and its potential polarization, we assessed the changes in cytokine expression profile from the PBMCs. As shown in Figure 3A, limited changes were observed in the cytokine profiles after each vaccine dose. In particular, no marked polarization was detected as Th1, Th2 and Th17 cytokines genes remained evenly expressed following vaccination. Analysis of Ig subclass expression profiles also indicated limited subclass switch following the first vaccine dose, and no major changes after subsequent doses (Figure 3B). Indeed, the first vaccine dose induced an increase in IgM, mostly at the expense of IgA expression, which was reduced, and this pattern was maintained after subsequent vaccine doses. Importantly, high levels of expression of IgG1 were sustained following vaccination, while no IgE was induced. Together, these results are in agreement with the induction of a broad and non-polarized immune response following vaccination, and three vaccine doses appear to be needed for a strong transcriptomic response of PBMCs.

We then typed the major histocompatibility complex (MHC) of the vaccinated macaques to assess potential MHC restriction of vaccine response. MHC class I alleles were the most diverse among the three macaques, with a total 6 and 5 alleles identified for Mamu A and Mamu B genes, respectively, while MHC class II genes were less diverse, with 1–4 alleles among these macaques (Table 1). As all three macaques developed an immune response to the vaccine antigens, these were adequately processed through these diverse MHC.

### Changes in immunoglobulin G repertoire following vaccination

**Figure 4:**
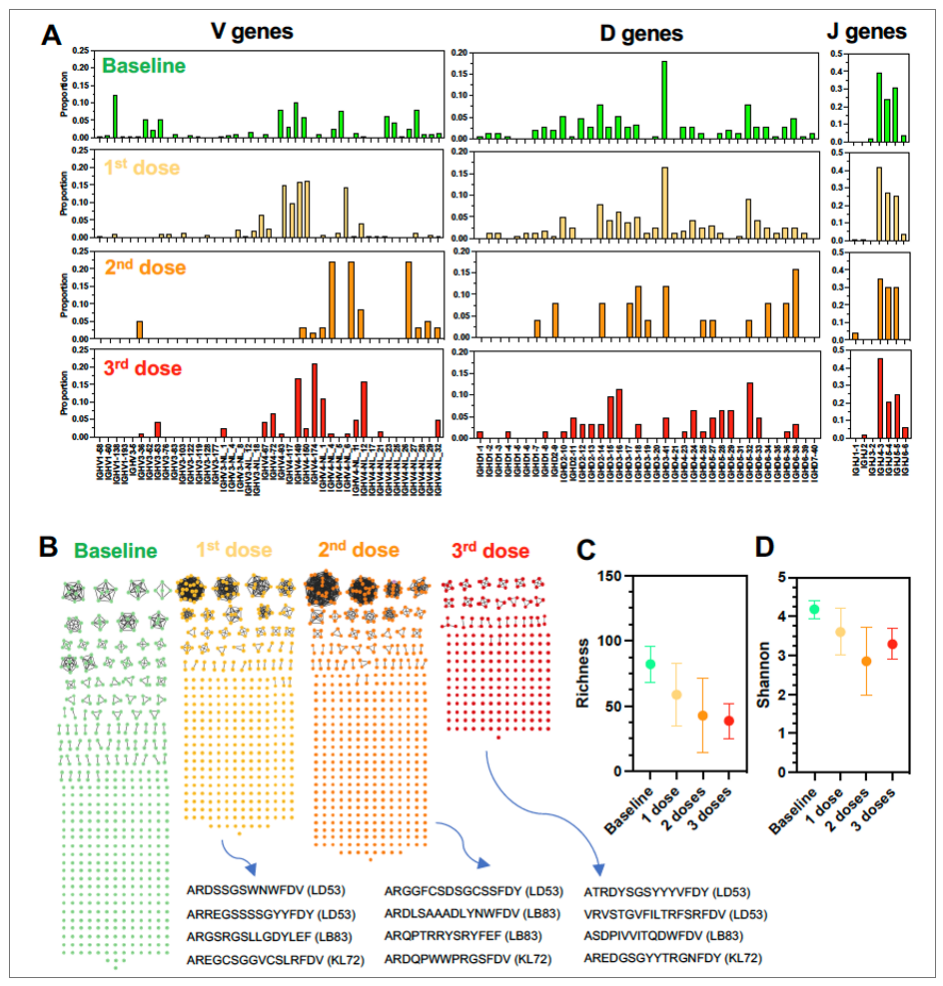
IgG repertoire following vaccination. **(A)** IgG VDJ gene usage frequency after vaccination of macaque KL72. **(B)** Network analysis of IgG CDR3 domains following each vaccine dose. Each circle node represents a unique CDR3 sequence, and edges link identical sequences. Color codes of the CDR3 nodes indicate sequences present at baseline and after each vaccine dose, respectively. Note that no CDR3 sequence persisted overtime, and that new sequences were present at each time-point. In each macaque, specific CDR3 sequences were particularly enriched after each vaccine dose, likely representing expansion of antigen-specific sequences (Bottom sequences with macaque ID indicated in parenthesis). **(C)** Richness S and **(D)** Shannon H diversity indices of CDR3 sequences over time and following vaccination (N=3 macaques).

Immunoglobulin G (IgG) diversity result from random combinations of variable (V), diversity (D), and joining (J) gene segments ^21,22^, to generate unique complementarity-determining region 3 (CDR3) within the hypervariable region of the protein which is involved in antigen-binding specificity ^23^. Thus, we assessed the changes in IgG repertoire and Ig heavy chain VDJ gene usage following vaccination. As shown in Figure 4A, changes in V and D gene usage could be detected after each vaccine dose, while J gene usage was mostly unaffected by vaccination. For example, IGHV1 and IGHD3 were the most frequently expressed genes prior to vaccination in macaque KL72, while after three vaccine doses IGHV4 genes were the most frequently used, as well as IGHD3 and IGHD5 genes (Figure 4A). Similar changes were detected in the other macaques. These data suggested that different IgGs are produced after each vaccine doses. Therefore, we analyzed Ig CDR3 domain sequence diversity and elaborated networks illustrating antibody repertoire of all three vaccinated macaques. At baseline, an extensive diversity of CDR3 sequences were observed, but most were uniques and only a few were present in more than one copy (Figure 4B). Following the first vaccine dose, a dramatic reduction in CDR3 sequence diversity was observed, while several sequences were much more abundant, likely indicative of the clonal expansion of vaccine antigen-specific antibodies (Figure 4B). For example, CDR3 sequences ARDSSGSWNWFDV, ARREGSSSSGYYFDY, ARGSRGSLLGDYLEF, AREGCSGGVCSLRFDV expanded after the first vaccine dose. Interestingly, no CDR3 sequence detected at baseline persisted after the first vaccine dose, and all CDR3 sequences were novel, in agreement with the changes in VD gene usage detected above in all macaques. After the second vaccine dose, all CDR3 sequences induced by the first vaccine dose were replaced by novel CDR3 sequences, indicating a major turnover of antibody-producing cell clones. CDR3 diversity remained limited compared to the baseline level, and new CDR3 sequences were further expanded. These included ARGGFCSDSGCSSFDY, ARDLSAAADLYNWFDV, ARQPTRRYSRYFEF, ARDQPWWPRGSFDV. This turn-over process appeared to be repeated after the third vaccine dose as well, and a new repertoire of CDR3 sequences was detected, although with more limited clonal expansion of CDR3 sequences (Figure 4B). Analysis of Richness (Figure 4C) and Shannon (Figure 4D) diversity indices over time confirmed that antibody diversity was reduced after the first and second vaccine dose, likely associated with the clonal expansion of a few vaccine antigen-specific IgGs. Although antibodies with novel CDR3 sequences were generated after the third vaccine dose, there were no further changes in the level of antibody diversity, suggesting that two vaccine doses may be sufficient for the induction of an optimum humoral response.

### Changes in T cell receptor repertoire following vaccination

**Figure 5:**
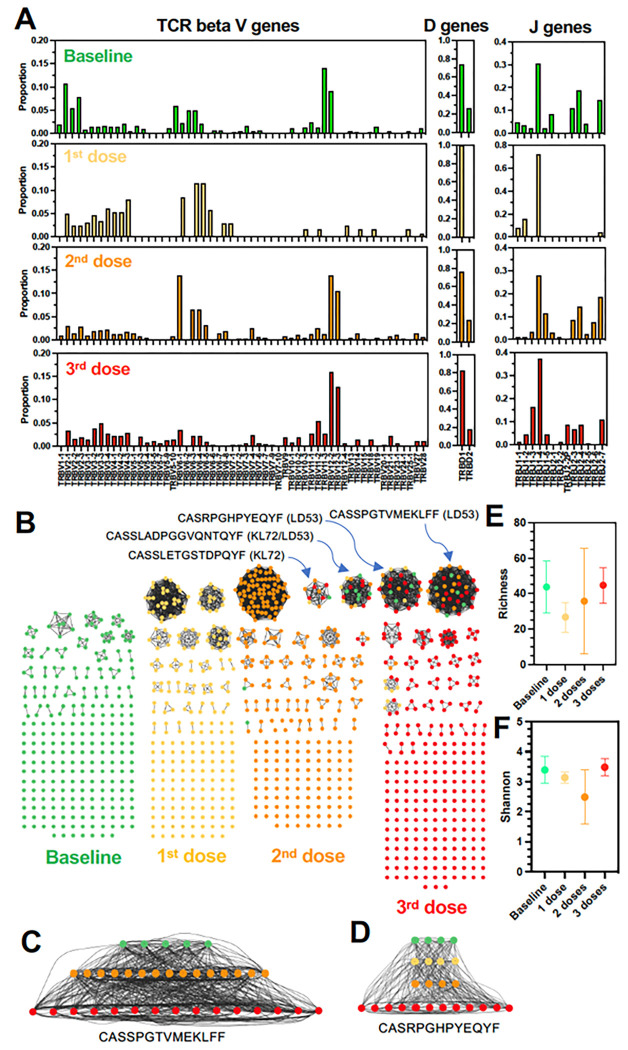
TCR beta repertoire following vaccination. **(A)** TCR beta VDJ gene usage frequency after vaccination of macaque LD53. **(B)** Network analysis of TCR beta CDR3 domains following each vaccine dose. Each circle node represents a unique CDR3 sequence, and edges link identical sequences. Color codes of the CDR3 nodes indicate sequences present at baseline and after each vaccine dose, respectively. Note that in addition of the identification of novel CDR3 sequences at each time-point following vaccination, some sequences present at baseline persisted and were enriched after each vaccine dose, likely representing clonal expansion of antigen-specific TCR sequences (Sequences indicated on top with macaque ID in parenthesis). **(C)** and **(D)** Magnified details of TCR beta CDR3 sequence networks illustrating their expansion after vaccination. **(E)** Richness S and **(F)** Shannon H diversity indices of CDR3 sequences over time and following vaccination (N=3 macaques).

T cell receptors (TCRs) are responsible for detecting epitopes presented by MHC molecules and their diversity is also driven by rearrangements of TCR beta V, D and J gene fragments and TCR alpha V and J genes, to generate unique CDR3 regions that determine TCR epitope binding affinity and specificity ^24,25^. Thus, TCR beta CDR3 diversity and VDJ gene usage were similarly analyzed. TRB VDJ gene usage resulted altered starting with the first vaccine dose, particularly for V and J genes (Figure 5A). For example, TRBV11 and TRBV12 genes frequently used at baseline were replaced by TRBV4 and TRBV6 which became the most used genes after the first vaccine dose in macaque LD53. These data are consistent with the induction of antigen-specific TCRs following vaccination. Further changes in gene usage were observed after the second vaccine dose, but limited changes seemed to occur after the third vaccine dose and TRBV12 and TRBJ1 genes were the most frequently used after vaccination.

Network analysis of TCR beta CDR3 domain diversity revealed further changes in the repertoire following vaccination (Figure 5B). Indeed, vaccination had limited effects on overall TCR beta CDR3 diversity, although it tended to decrease after the first and second dose of vaccine as assessed by Richness and Shannon indices (Figure 5E and F). Nonetheless, novel CDR3 sequences were induced after each vaccine dose and several. were present at high frequencies, likely representing antigen-specific TCR sequences. Moore strikingly, a few sequences present a low frequency at baseline persisted and were present at increasing frequencies following each vaccine dose, including the third dose (Figure 5B, C and D). These corresponded to CDR3 sequences CASSPGTVMEKLFF, CASRPGHPYEQYF, CASSLADPGGVQNTQYF, or CASSLETGSTDPQYF, for example. One of these CDR3 sequence was also identified in two macaques (CASSLADPGGVQNTQYF, detected in macaques KL72 and LD53), indicating convergence of their immune response. These data evidenced a strong T cell response to vaccination, with the likely proliferation of cells with antigen-specific TCR beta.

## DISCUSSION

The development of a Chagas disease vaccine would be a key step towards a better control of this neglected disease, and a vaccine prototype based on Tc24 and TSA1 antigens is emerging as an attractive candidate for further development ^10,13^. An initial evaluation of this vaccine in naïve macaque suggested that it could stimulate both B and T cell immunity ^16^. We aimed here to expand these observations by assessing the transcriptomic response of PBMCs from vaccinated macaques, and assess changes in their IgG and TCR repertoires.

Transcriptomic analysis revealed that no changes in gene expression profiles were detected one month after the first vaccine dose, and only marginal changes one month after the second dose, so that three vaccine doses were required to detect significant alterations in the PBMC gene expression profile. This was somewhat unexpected as a large increase in antigen-specific IgG could already be detected one month after the first vaccine dose, indicating immunogenicity at this early time point ^16^. However, the response to vaccines is largely asynchronous in humans, with early and delayed responses depending on the vaccine and its formulation ^26^. Similarly, the transcriptomic response of PBMCs from macaques vaccinated with BCG is larger on day two after vaccination and decreases in the following weeks ^27^. On the other hand, PBMCs from humans vaccinated with *Plasmodium falciparum* sporozoites present a transcriptomic response at day 27 post-immunization but not before ^28^. Thus, the inclusion of additional time points would be needed for a fine scale analysis of the kinetics of transcriptomic changes to this Chagas disease vaccine, as it is unclear what changes may occur in the few days after immunization. Also, increasing sample size would also allow to reach a greater power to identify differentially expressed genes as we observed many genes presenting potential changes in expression levels after the first vaccine dose, but none reached statistical significance.

On the other hand, there was a clear transcriptomic response of PBMCs after the third vaccine dose, with over 600 differentially expressed genes, confirming its immunogenicity. These genes were involved in several metabolic/catabolic functions which appeared down regulated, suggesting changes in cell activity/differentiation. Also, as expected, several pathways associated with the activation of immune cells from leukocytes to T cells were upregulated, indicative of an active immune response. The immunoglobin expression profile indicated limited subclass switch, as the main change was an increase in the proportion of IgM while IgA was reduced, and the other subclasses remained unaltered. In particular, the production of IgG1 remained predominant among IgGs, and this isotype is associated with the highest effector function activity in macaques ^29^. Notably, the expression of IgE, which is associated with allergic reactions ^30^ was negligible, suggesting a lack of allergic reaction to the vaccine, although the analysis of nasal secretion would be required for confirmation ^31^. Analysis of the cytokine expression profile also suggested the induction of a balanced immune response, including Th1, Th2 and Th17 cytokines. Such a balanced immune response would be indicated for an effective vaccine against *T. cruzi*, as a cellular response is critical for parasite control but hyperpolarization may lead to tissue damage ^10,32^. These data support the further evaluation of vaccine efficacy against *T. cruzi* infection.

Analysis of the IgG heavy chain repertoire indicated changes in their VDJ gene usage one month after the first vaccine dose, and after each following vaccine dose. These changes were associated with a renewal of the CDR3 repertoire after each vaccine dose, and no clone persisted over time. Rapid changes in Ig CDR3 repertoire have similarly been observed at 7 and 28 days post-vaccination with a pneumococcal vaccine in humans ^33^. Also, the longitudinal follow-up of the human IgG repertoire showed that >85% of CDR3s can be detected only once out of 24 time points over a 11 months period ^34^, indicating a very rapid turnover. Although vaccine antigens Tc24 and TSA1 are both highly conserved among *T. cruzi* strains ^35,36^, the generation of diverse polyclonal antibodies against them may help broaden their binding affinity to accommodate for more sequence variants and/or increase their efficacy by targeting different regions of the antigens.

Vaccination also induced important changes in T cell receptor repertoire, with the notable persistence and expansion of specific CDR3 sequences over at least 4 months, as well as some convergence in two of the vaccinated macaques. These expanded CDR3 clones are likely antigen specific and further studies should help assess this specificity. These data evidence a clear T cell response to vaccination and are in agreement with the activation of T cells targeting multiple epitopes from the vaccine antigens, and their expansion over at least 4 months suggest the presence of memory T cells. Vaccination in humans has often been associated with increases in TCR beta CDR3 diversity, for example in response to a Rabies virus vaccine ^37^ or a Hepatitis B vaccine ^38^, but our data suggest a more focused response to our vaccine candidate with no expansion of the TCR repertoire. This may be due to differences among vaccine antigens and their immune processing, or differences in the kinetics of these responses as more time points would be needed for a more detailed analysis of their time course, as mentioned above. Also, while two vaccine doses were sufficient to induce changes in TCR repertoire and expansion of some CDR3 clones, the third vaccine dose brought further expansion of these clones and may thus be needed for a stronger immunogenicity of this vaccine. Future studies should help assess potential differences in protective efficacy after two or three vaccine doses.

Remarkably, all three vaccinated macaques presented transcriptomic responses and changes in their IgG and TCR repertoires following vaccination, in the context of a varied MHC background, particularly for class I MHC. This is also encouraging as it suggests that so far there are no MHC restrictions of vaccine immunogenicity ^39,40^ and future studies should include animals with additional MHC background to broaden this evaluation.

In conclusion, we identified here a clear transcriptomic response of PBMCs from macaques vaccinated a Chagas disease vaccine based on Tc24 and TSA1 parasite antigens, with the up-regulation of several immune cell activation pathways. These data confirming the immunogenicity of this vaccine, with a broad non-polarized immune profile. While changes in IgG and TCR repertoires could be detected one month after the first vaccine dose, further changes were observed after the second and third vaccine dose. The IgG repertoire showed a rapid turnover with new IgGs following each vaccine dose, while the TCR repertoire presented persisting clones that were expanded after each vaccine dose, suggesting that three vaccine doses may be needed for optimum efficacy. This work warrants the further characterization of the IgG and T cell receptor repertoires of Chagas disease patients ^41^, particularly with different stages of disease progression/parasite control, to identify immune repertoire profiles associated with better disease outcomes and correlates for protection. Also, the evaluation of the protective efficacy of this vaccine candidate against *T. cruzi* infection will be key for vaccine development and the availability of naturally-infected macaques provide an ideal model for testing a therapeutic vaccine ^42^.

## METHODS

### Animals and vaccination

Animals were housed at the Tulane National Primate Research Center (TNPRC) under the care of TNPRC veterinarians, in accordance with the standards incorporated in the Guide for the Care and Use of Laboratory Animals and with the approval of Tulane Institutional Animal Care and Use Committee (IACUC). Three naïve male rhesus macaques (*Macaca mulatta*) 4–5 years old were vaccinated with three doses of the vaccine based on Tc24-C4 and TSA1-C4 antigens formulated with a TLR4 adjuvant, one month apart, as described before ^16^. A blood sample was collected in EDTA at baseline and one month after each vaccine dose. PBMCs were isolated and cryopreserved until used for RNA purification and RNA-sequencing.

### RNA purification and RNA sequencing

Cryopreserved PBMCs ^16^ were checked for viability with trypan blue, and about 10^6^ cells were used for RNA extraction using PerfectPure RNA Cultured Cell Kitä (5 Prime, Inc.) following manufacturer instructions. RNA integrity was assessed on an Agilent BioAnalyzer and all samples had a RIN>8. About 200 ng of RNA was used for library preparation and sequencing on an Illumina MiSeq platform, and about 12–35 million reads/sample were obtained after quality filtering. Raw reads have been deposited in NCBI SRA database under Bioproject #PRJNA1010169, Biosamples SAMN37182435-SAMN37182446.

### Transcription profile

Reads were mapped to the Rhesus macaque reference genome (Mmul10 accession: GCF_003339765.1) in Geneious 11. Read counts were then normalized and differentially expressed genes called using DESeq2 as implemented in iDEP1.1 ^43^. One sample was removed from further analysis (macaque KL72, second vaccine dose) due to a very low number of reads. Differences in gene expression levels >1.5 fold change were called at a significance alpha of 0.05 adjusted for multiple testing using the false discovery rate method. Volcano plots were used to visualize differentially expressed genes. Enriched functional pathway associated with up-regulated and down-regulated genes, respectively, were identified using ShinyGO 0.77 ^44^, based on Gene Ontology Biological Process database.

### MHC typing

RNA-seq read counts mapping to MHC reference genes (Mamu A, Mamu B, Mamu DPA, Mamu DPB, Mamu DQA, Mamu DQB and Mamu DRA) for individual macaques were used for de-novo assembly in Geneious. Assembled MHC sequences were compared with the IDP-MHC allele database ^45^ using BLAST and the top two matches with >98% sequence identity for each MHC gene were retained.

### Antibody isotype

RNA-seq read counts mapping to unique 150 bp regions of IgA, IgE, IgG1, IgG2, IgG3, IgG4 and IgM subclasses were used to assess the relative expression level of the respective antibody isotype flowing vaccination. These were normalized according to the total number of reads mapping to the macaque genome from each sample, to account for differences in sequencing depth and coverage and provide relative abundance level of Ig isotypes.

### IgG antibody repertoire

Reads mapping to the variable CDR3 region of IgG heavy chain gene, which mediates antibody specificity, as well as flanking regions were extracted for analysis of IgG variable domain repertoire using IgBLAST ^46^. The combination of the variable (V) gene, the diversity (D) gene and the joining (J) gene from each sequence was determined to assess gene usage frequency for IgG production in individual macaques following vaccination. The translated CDR3 sequences were analyzed for frequency and similarity using the EFI Enzyme Similarity Tool ^47^ and similarity networks were elaborated in Cytoscape to visualize changes in antibody repertoire over time. CDR3 sequence diversity was further assessed using Shannon H and Richness S summary indices, which were calculated in Past4.0 ^48^.

### T cell receptor repertoire

Reads mapping to the CDR3 region of the TCR beta subunit gene, which mediates T cell epitope binding specificity, as well as flanking regions were extracted for analysis of TCR variable domain repertoire using IMGT/HighV-QUEST ^49,50^. The combination of the TCR beta V/D/J genes from each sequence was determined to assess TCR gene usage frequency and its changes following vaccination. The translated CDR3 sequences were analyzed for frequency, similarity and diversity as described above.

## Figures and Tables

**Table 1.** MHC typing of vaccinated macaques

Macaque ID	KL72		LB83		LD53	
	Allele 1	Allele 2	Allele 1	Allele 2	Allele 1	Allele 2
Mamu A	A3*13	A1*008	A6:01	A1*003	A4*14	A1*004
Mamu B	B*052	B*072	B*060	B*024	B*002	B*052
Mamu DPA	DPA1*04	DPA1*04	DPA1*04	DPA1*04	DPA1*02	DPA1*02
Mamu DPB	DPB1*02	DPB1*02	DPB1*02	DPB1*03	DPB1*07	DPB1*15
Mamu DQA	DQA1*24	DQA1*26	DQA1*24	DQA1*24	DQA1*23	DQA1*26
Mamu DQB	DQB1*15	DQB1*18	DQB1*15	DQB1*18	DQB1*18	DQB1*18
Mamu DRA	DRA*01	DRA*01	DRA*01	DRA*01	DRA*01	DRA*01

## Data Availability

The datasets generated and/or analyzed during the current study are available in the NCBI SRA database under Bioproject #PRJNA1010169, Biosamples SAMN37182435-SAMN37182446.

## References

[1] WHO. Chagas disease in Latin America: an epidemiological update based on 2010 estimates. Weekly epidemiological record 90, 33–43 (2015). 25671846

[2] Lee B. Y., Bacon K. M., Bottazzi M. E. & Hotez P. J. Global economic burden of Chagas disease: a computational simulation model. The Lancet infectious diseases 13, 342–348 (2013). 10.1016/S1473-3099(13)70002-1 23395248 PMC3763184

[3] Rassi A. Jr., Rassi A. & Marin-Neto J. A. Chagas disease. Lancet 375, 1388–1402 (2010). https://doi.org/S0140-6736(10)60061-X [pii] 20399979 10.1016/S0140-6736(10)60061-X

[4] Pinazo M. J. Tolerance of benznidazole in treatment of Chagas’ disease in adults. Antimicrobial agents and chemotherapy 54, 4896–4899 (2010). 10.1128/AAC.00537-10 20823286 PMC2976114

[5] Morillo C. A. Randomized Trial of Benznidazole for Chronic Chagas’ Cardiomyopathy. The New England journal of medicine 373, 1295–1306 (2015). 10.1056/NEJMoa1507574 26323937

[6] Castro J. A., de Mecca M. M. & Bartel L. C. Toxic side effects of drugs used to treat Chagas’ disease (American trypanosomiasis). Hum Exp Toxicol 25, 471–479 (2006). 16937919 10.1191/0960327106het653oa

[7] Forsyth C. Proposed multidimensional framework for understanding Chagas disease healthcare barriers in the United States. PLoS neglected tropical diseases 13, e0007447 (2019). 10.1371/journal.pntd.0007447 31557155 PMC6762052

[8] Hotez P. J. Chagas disease: “the new HIV/AIDS of the Americas”. PLoS neglected tropical diseases 6, e1498 (2012). 10.1371/journal.pntd.0001498 22666504 PMC3362306

[9] Proano A., Dumonteil E. & Herrera C. Chagas Disease Diagnostic Testing in Two Academic Hospitals in New Orleans, Louisiana: A Call to Action. Trop Med Infect Dis 8 (2023). 10.3390/tropicalmed8050277 PMC10223931 37235325

[10] Dumonteil E. & Herrera C. The Case for the Development of a Chagas Disease Vaccine: Why? How? When? Trop Med Infect Dis 6 (2021). 10.3390/tropicalmed6010016 PMC7851737 33530605

[11] Dumonteil E. Vaccine development against *Trypanosoma cruzi* and *Leishmania* species in the post-genomic era. Infection, genetics and evolution : journal of molecular epidemiology and evolutionary genetics in infectious diseases 9, 707–711 (2009). 10.1016/j.meegid.2009.02.009 19805015

[12] Dumonteil E., Herrera C. & Marx P. A. Safety and preservation of cardiac function following therapeutic vaccination against *Trypanosoma cruzi* in rhesus macaques. J Microbiol Immunol Infect 56, 400–407 (2023). 10.1016/j.jmii.2022.09.003 36210315 PMC10131272

[13] Dumonteil E. Accelerating the development of a therapeutic vaccine for human Chagas disease: rationale and prospects. Expert review of vaccines 11, 1043–1055 (2012). 10.1586/erv.12.85 23151163 PMC3819810

[14] Martinez-Campos V. Expression, purification, immunogenicity, and protective efficacy of a recombinant Tc24 antigen as a vaccine against *Trypanosoma cruzi* infection in mice. Vaccine 33, 4505–4512 (2015). 10.1016/j.vaccine.2015.07.017 26192358

[15] de la Cruz J. J. Production of recombinant TSA-1 and evaluation of its potential for the immuno-therapeutic control of Trypanosoma cruzi infection in mice. Human vaccines & immunotherapeutics 1 5, 210–219 (2019). 10.1080/21645515.2018.1520581 PMC6363145 30192702

[16] Dumonteil E. Safety and immunogenicity of a recombinant vaccine against *Trypanosoma cruzi* in Rhesus macaques. Vaccine 38, 4584–4591 (2020). 10.1016/j.vaccine.2020.05.010 32417142 PMC7310587

[17] Georgiou G. The promise and challenge of high-throughput sequencing of the antibody repertoire. Nat Biotechnol 32, 158–168 (2014). 10.1038/nbt.2782 24441474 PMC4113560

[18] Jiang N. Lineage structure of the human antibody repertoire in response to influenza vaccination. Sci Transl Med 5, 171ra119 (2013). 10.1126/scitranslmed.3004794 PMC3699344 23390249

[19] Gong Q. Assessment of T-cell receptor repertoire and clonal expansion in peripheral T-cell lymphoma using RNA-seq data. Sci Rep 7, 11301 (2017). 10.1038/s41598-017-11310-0 28900149 PMC5595876

[20] Shao M. M. T Cell Receptor Repertoire Analysis Reveals Signatures of T Cell Responses to Human Mycobacterium tuberculosis. Front Microbiol 13, 829694 (2022). 10.3389/fmicb.2022.829694 35197957 PMC8859175

[21] DeKosky B. J. Large-scale sequence and structural comparisons of human naive and antigen-experienced antibody repertoires. Proceedings of the National Academy of Sciences of the United States of America 113, E2636–2645 (2016). 10.1073/pnas.1525510113 27114511 PMC4868480

[22] Mroczek E. S. Differences in the composition of the human antibody repertoire by B cell subsets in the blood. Front Immunol 5, 96 (2014). 10.3389/fimmu.2014.00096 24678310 PMC3958703

[23] Xu J. L. & Davis M. M. Diversity in the CDR3 region of V(H) is sufficient for most antibody specificities. Immunity 13, 37–45 (2000). 10.1016/s1074-7613(00)00006-6 10933393

[24] Mariuzza R. A., Agnihotri P. & Orban J. The structural basis of T-cell receptor (TCR) activation: An enduring enigma. J Biol Chem 295, 914–925 (2020). 10.1074/jbc.REV119.009411 31848223 PMC6983839

[25] Garcia K. C. & Adams E. J. How the T cell receptor sees antigen--a structural view. Cell 122, 333–336 (2005). 10.1016/j.cell.2005.07.015 16096054

[26] Hagan T. Transcriptional atlas of the human immune response to 13 vaccines reveals a common predictor of vaccine-induced antibody responses. Nat Immunol 23, 1788–1798 (2022). 10.1038/s41590-022-01328-6 36316475 PMC9869360

[27] Liu Y. E. Blood transcriptional correlates of BCG-induced protection against tuberculosis in rhesus macaques. Cell Rep Med, 101096 (2023). 10.1016/j.xcrm.2023.101096 37390827 PMC10394165

[28] Tran T. M. Whole-blood transcriptomic signatures induced during immunization by chloroquine prophylaxis and Plasmodium falciparum sporozoites. Sci Rep 9, 8386 (2019). 10.1038/s41598-019-44924-7 31182757 PMC6557840

[29] Boesch A. W. Biophysical and Functional Characterization of Rhesus Macaque IgG Subclasses. Front Immunol 7, 589 (2016). 10.3389/fimmu.2016.00589 28018355 PMC5153528

[30] Gould H. J. & Sutton B. J. IgE in allergy and asthma today. Nat Rev Immunol 8, 205–217 (2008). 10.1038/nri2273 18301424

[31] Nakayama M. Potential risk of repeated nasal vaccination that induces allergic reaction with mucosal IgE and airway eosinophilic infiltration in cynomolgus macaques infected with H5N1 highly pathogenic avian influenza virus. Vaccine 35, 1008–1017 (2017). 10.1016/j.vaccine.2017.01.008 28109707

[32] Jones K. M., Poveda C., Versteeg L., Bottazzi M. E. & Hotez P. J. Preclinical advances and the immunophysiology of a new therapeutic Chagas disease vaccine. Expert review of vaccines 21, 1185–1203 (2022). 10.1080/14760584.2022.2093721 35735065

[33] Ademokun A. Vaccination-induced changes in human B-cell repertoire and pneumococcal IgM and IgA antibody at different ages. Aging Cell 10, 922–930 (2011). 10.1111/j.1474-9726.2011.00732.x 21726404 PMC3264704

[34] Mitsunaga E. M. & Snyder M. P. Deep Characterization of the Human Antibody Response to Natural Infection Using Longitudinal Immune Repertoire Sequencing. Molecular & cellular proteomics : MCP 19, 278–293 (2020). 10.1074/mcp.RA119.001633 31767621 PMC7000125

[35] Arnal A., Villanueva-Lizama L., Teh-Poot C., Herrera C. & Dumonteil E. Extent of polymorphism and selection pressure on the Trypanosoma cruzi vaccine candidate antigen Tc24. Evol Applications 13, 2663–2672 (2020). 10.1111/eva.13068 PMC7691455 33294015

[36] Knight J. M., Zingales B., Bottazzi M. E., Hotez P. & Zhan B. Limited antigenic variation in the *Trypanosoma cruzi* candidate vaccine antigen TSA-1. Parasite immunology 36, 708–712 (2014). 10.1111/pim.12130 25040249

[37] Zhao P. Quantitative characterization of the T cell receptor repertoires of human immunized by rabies virus vaccine. Human vaccines & immunotherapeutics 17, 2530–2537 (2021). 10.1080/21645515.2021.1893575 33823121 PMC8475554

[38] Miyasaka A., Yoshida Y., Wang T. & Takikawa Y. Next-generation sequencing analysis of the human T-cell and B-cell receptor repertoire diversity before and after hepatitis B vaccination. Human vaccines & immunotherapeutics 15, 2738–2753 (2019). 10.1080/21645515.2019.1600987 30945971 PMC6930056

[39] Ovsyannikova I. G., Dhiman N., Jacobson R. M. & Poland G. A. Human leukocyte antigen polymorphisms: variable humoral immune responses to viral vaccines. Expert review of vaccines 5, 33–43 (2006). 10.1586/14760584.5.1.33 16451106

[40] Poland G. A., Ovsyannikova I. G., Jacobson R. M. & Smith D. I. Heterogeneity in vaccine immune response: the role of immunogenetics and the emerging field of vaccinomics. Clin Pharmacol Ther 82, 653–664 (2007). 10.1038/sj.clpt.6100415 17971814

[41] de Souza-Silva T. G., Gollob K. J. & Dutra W. O. T-cell receptor variable region usage in Chagas disease: A systematic review of experimental and human studies. PLoS neglected tropical diseases 16, e0010546 (2022). 10.1371/journal.pntd.0010546 36107855 PMC9477334

[42] Dumonteil E. Intra-host Trypanosoma cruzi strain dynamics shape disease progression: the missing link in Chagas disease pathogenesis. Microbiol Spectr (2023) in press. 10.1128/spectrum.04236-22 PMC10581044 37668388

[43] Ge S. X., Son E. W. & Yao R. iDEP: an integrated web application for differential expression and pathway analysis of RNA-Seq data. BMC bioinformatics 19, 534 (2018). 10.1186/s12859-018-2486-6 30567491 PMC6299935

[44] Ge S. X., Jung D. & Yao R. ShinyGO: a graphical gene-set enrichment tool for animals and plants. Bioinformatics 36, 2628–2629 (2020). 10.1093/bioinformatics/btz931 31882993 PMC7178415

[45] Barker D. J. The IPD-IMGT/HLA Database. Nucleic acids research 51, D1053–D1060 (2023). 10.1093/nar/gkac1011 36350643 PMC9825470

[46] Ye J., Ma N., Madden T. L. & Ostell J. M. IgBLAST: an immunoglobulin variable domain sequence analysis tool. Nucleic acids research 41, W34–40 (2013). 10.1093/nar/gkt382 23671333 PMC3692102

[47] Oberg N., Zallot R. & Gerlt J. A. EFI-EST, EFI-GNT, and EFI-CGFP: Enzyme Function Initiative (EFI) Web Resource for Genomic Enzymology Tools. J Mol Biol 435, 168018 (2023). 10.1016/j.jmb.2023.168018 37356897 PMC10291204

[48] Hammer Ø., Harper D. A. T. & Ryan P. D. PAST: Paleontological statistics software package for education and data analysis. Palaeontologia Electronica 4, 9pp (2001).

[49] Li S. IMGT/HighV QUEST paradigm for T cell receptor IMGT clonotype diversity and next generation repertoire immunoprofiling. Nat Commun 4, 2333 (2013). 10.1038/ncomms3333 23995877 PMC3778833

[50] Alamyar E., Duroux P., Lefranc M. P. & Giudicelli V. IMGT((R)) tools for the nucleotide analysis of immunoglobulin (IG) and T cell receptor (TR) V-(D)-J repertoires, polymorphisms, and IG mutations: IMGT/V-QUEST and IMGT/HighV-QUEST for NGS. Methods Mol Biol 882, 569–604 (2012). 10.1007/978-1-61779-842-9_32 22665256

